# Supplementary material for: Angelica sinensis polysaccharide as potential protectants against recurrent spontaneous abortion: focus on autophagy regulation
Source: Front Med (Lausanne). 2025 Jan 15;12:1522503. doi: 10.3389/fmed.2025.1522503 (PMC11774876; doi:10.3389/fmed.2025.1522503)
Supplement: Supplementary material S1 — The main instruments used during the LC-MS process, along with their models/specifications and manufacturers. [file Supplementary_file_1.zip › Metabolomics sequencing data FC1.2/4.多元统计分析/Splot/Splot-OPLS-DA(Exposure_Ctrl).pdf]

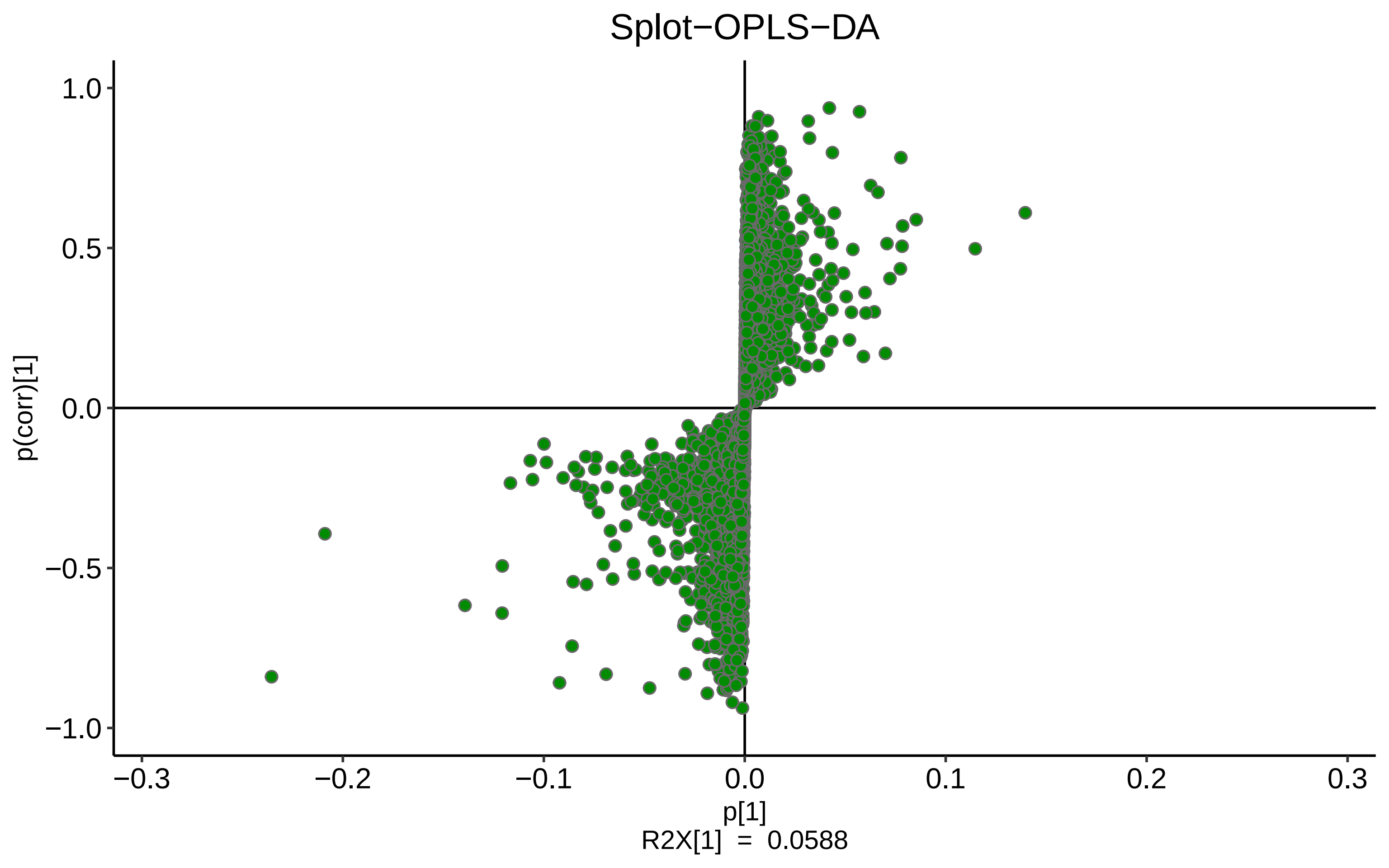
Splot−OPLS−DA
p(corr)[1]
1.0
0.5
0.0
−0.5
−1.0
−0.3
−0.2
−0.1
0.0
0.1
0.2
0.3
p[1]
R2X[1] = 0.0588
